# Mapping the Human Performance Envelope Through Multivariate Information Transfer

**DOI:** 10.3390/brainsci16050518

**Published:** 2026-05-13

**Authors:** Gianluca Borghini, Khadija Latrach, Gianluca Di Flumeri, Pietro Aricò, Vincenzo Ronca, Andrea Giorgi, Rossella Capotorto, Alessia Ricci, Stefano Bonelli, Vanessa Arrigoni, Paola Tomasello, Fabrice Drogoul, Jean Paul Imbert, Géraud Granger, Fabio Babiloni

**Affiliations:** 1 Department of Molecular Medicine, Sapienza University of Rome, Piazzale Aldo Moro, 5, 00185 Rome, Italy; 2 Department of Computer, Control, and Management Engineering Antonio Ruberti, Sapienza University of Rome, Piazzale Aldo Moro, 5, 00185 Rome, Italy; 3 Department of Anatomical, Histological, Forensic & Orthopedic Sciences, Sapienza University of Rome, Piazzale Aldo Moro, 5, 00185 Rome, Italy; 4 DeepBlue srl, Piazza Buenos Aires 20, 00185 Rome, Italy; 5 EUROCONTROL, Rue de la Fusée 96, 1130 Brussels, Belgium; 6 Aeronautical Computer Human Interaction Lab (ACHIL), École Nationale de l’Aviation Civile, 7 Avenue Edouard Belin, 31000 Toulouse, France; 7 Department of Physiology and Pharmacology “Vittorio Erspamer”, Sapienza University of Rome, Piazzale Aldo Moro, 5, 00185 Rome, Italy; 8 College of Computer Science and Technology, Hangzhou Dianzi University, 115 Wenyi Rd, Xihu, Hangzhou 310005, China

**Keywords:** human performance envelope (HPE), neurophysiological, mental states, conditional transfer entropy (cTE), Least Absolute Shrinkage and Selection Operator (LASSO), human factors (HF), air traffic controller (ATCO)

## Abstract

**Highlights:**

**What are the main findings?**

- The human performance envelope (HPE) can be objectively quantified as the dynamic, polygonal area defined by the causal interactions of five core neurophysiological human factors: mental workload, attention, stress, vigilance, and effort.
- Optimal performance states (best) are characterized by a significantly larger HPE area and a denser, more interconnected network of human factors, whereas low-performance states (worst) exhibit a contracted HPE area and fragmented connectivity.

**What are the implications of the main findings?**

- The LASSO-regularized MVAR-cTE pipeline provides the first directed, multivariate neurophysiological model of the HPE, bridging a critical gap between theoretical concepts and operational human states and performance assessment.
- The finding that operational resilience is underpinned by integrated neurocognitive networks offers a new biomarker for adaptive system design and competency-based training in safety-critical domains.

**Abstract:**

Background/Objectives: The human performance envelope (HPE) is a multidimensional model that represents the range in which an individual operator’s performance is acceptable or begins to become dangerous. Although several alternative models have been proposed, HPE currently remains primarily a theoretical concept. The goal of the study was therefore to translate this theoretical concept into practical applications, seeking to characterize and measure how HPE manifests itself in real-world contexts. Methods: Multivariate Autoregressive (MVAR) models and conditional transfer entropy (cTE) have been used in the analysis of complex systems in which processes are interdependent and mutually influence their dynamics over time. Professional Air Traffic Controllers were involved in the study and asked to deal with realistic traffic scenarios while their behavioural, subjective and neurophysiological data were collected. MVAR–cTE models were then employed to estimate the interactions among controller human factors and to identify the most appropriate characterization of the HPE. Results: The results showed high and significant correlations among each controller’s performance and the corresponding neurophysiological-based HPE values. Furthermore, high-performance conditions (best) were characterized by significantly higher HPE values and higher inter-human factor connections compared to the low-performance (worst) status. This evidence suggested that a densely interconnected network of Human Factors is a prerequisite for operational resilience. Conclusions: The study provided the first application of a neurophysiological framework to model the directed interactions between human factors, translating the theoretical HPE into a quantifiable model validated against operator performance.

## 1. Introduction

The relentless evolution of aviation, marked by increasing traffic density, the introduction of trajectory-based operations, and a fundamental shift towards closer human–automation collaboration, propels air traffic management (ATM) into an era of unprecedented complexity [1,2,3]. While technology advancements in decision-support tools offer remarkable capabilities, the ultimate guarantor of system safety remains the air traffic controller (ATCO). This reality compels a critical scientific and operational focus on the human performance envelope (HPE). The HPE is a psycho-behavioural concept introduced by Bittner in 1985 [4] with the goal of improving the evaluation of the individual operator. In particular, the HPE is a multidimensional theoretical model where human factors (HFs) define the region in which the operator’s performance is acceptable or when the situation becomes dangerous. Depending on the combinations of different HFs, the resulting HPE could move from optimal to degraded [5,6]. For example, when a single HF shows an unacceptable value (e.g., high mental workload), its interaction with all others can compensate it and the overall performance can remain acceptable. Operating within this envelope allows for the graceful management of routine traffic and the adaptive, resilient handling of anomalies. Exceeding the HPE’s boundaries precipitates a degradation in performance, a phenomenon characterized by errors of omission (inattention) or commission (misjudgement), loss of situation awareness, and ultimately, a compromise in system safety margins.

This example suggests that HPE cannot simply be modelled as a linear combination of HFs, but that it requires more sophisticated and detailed modelling. Contemporary research has in fact profoundly evolved the understanding of the HPE from a simple, scalar measure of “workload” to a dynamic, multi-layered, and adaptive system [7,8]. The HPE is therefore shaped by a host of intrinsic and extrinsic factors like expertise, circadian chronotype, trait anxiety, sleep history, and even genetic predispositions [9]. Additionally, cognitive processes are underpinned by a physiological substrate that imposes its own boundaries. These boundaries are elastic and state-dependent. Indeed, factors like motivation, time-on-task (fatigue), and perceived risk can expand or contract it momentarily [10]. Furthermore, performance degradation is often non-linear, and a system may appear stable until a critical threshold is crossed, leading to a rapid, cliff-edge decline—a phenomenon poorly captured by traditional linear models. It is therefore clear how the concept of HPE is crucial in several high-stakes fields such as Aviation, Space, Transport, Medicine, and Defence. It encompasses the range of conditions under which a human can perform tasks effectively and safely. Understanding the HPE is hence vital for optimizing human performance, enhancing safety, and designing systems that support human operators.

Over the years, numerous studies have aimed at transforming the theoretical HPE model into indicators. One of the most significant attempts is represented by the study conducted by Alina-Ioana Chira [11]. However, despite extensive efforts, this study has been limited to reporting the values of different and single neurometrics during various phases of experimentation, without modelling the interactions of different HFs. A similar approach has been adopted by Victoria Rusu and Gavrila Calefariu [12]. In this case, the focus was on physiological parameters, such as blood pressure and blood oxygen level. Although such studies have represented a significant step forward in the field of research, they did not succeed in providing a complete model for HPE that accurately predicted individuals’ performance based on the interactions between HFs. A recent study conducted by Graziani [13] delved into an analysis of HFs within the realm of HPE. Initially, the investigation focused on identifying and evaluating the various HFs involved in the study. The article aimed to explore the potential of human–machine interaction as a tool to facilitate recovery and enhance the performance of the subjects under examination. Through careful and targeted analysis, the focus of the study gradually shifted from HPE to human–machine interaction as the primary objective for optimizing human performance. Therefore, the article highlighted not only the importance of understanding HFs but also developing targeted strategies for human–machine interaction to maximize human potential and improve overall performance. Another significant contribution in the field of human performance enhancement emerged through the study conducted by Biella [14]. This work was centred on the application of eye tracking as a key analytical tool. The primary goal of the research was to investigate the relationship between subjects’ gaze fixation points and their performance in various activities. This innovative approach opened new perspectives in human performance enhancement, providing a detailed and dynamic analysis of visual engagement during specific tasks. Identifying how visual focus directly influences individuals’ executive abilities marked a significant step forward in defining human performance enhancement. Another noteworthy study of Dimitrescu et al. [15] introduced an innovative approach to the analysis of human performance. Within the framework of this study, a device capable of detecting subjects’ emotions through heart rate measurement was developed. Through the implementation of this technology, researchers were able to closely examine participants’ emotional responses during specific tasks. One of the primary objectives was to assess how emotions influenced individual performance in operational contexts.

A fundamental limitation shared by all these approaches to HPE characterization is their reliance on pairwise (i.e., relationships between pairs of processes) or univariate indices.

In a complex operational system such as air traffic control, the cognitive state of an operator is shaped by the simultaneous interaction of multiple HFs whose dynamics are mutually interdependent over time. Bivariate or univariate methods are structurally unable to account for this interdependence: an apparent directed influence between two HFs may in fact be an indirect effect mediated by a third HF or may reflect shared common input rather than a genuine direct connection [16]. Consequently, analyzing HFs in isolation or in pairs fundamentally risks misrepresenting the functional architecture of the multivariate system that underlies operator performance.

To address this limitation, a genuinely multivariate approach is required, one that estimates directed connections between HFs while simultaneously conditioning on the full context of all other HFs in the system. Conditional transfer entropy (cTE) provides exactly this capability: by conditioning the information transfer from a source HF to a target HF on the past history of all remaining HFs simultaneously, cTE filters out spurious or indirect influences and identifies only direct predictive dependencies [16,17,18]. This level of multivariate precision is essential for HPE characterization, as it ensures that the resulting model reflects the actual coordination structure between mental states rather than statistical artefacts of bivariate analysis. However, estimating such a fully multivariate network in real-world scenarios presents a significant data-paucity challenge. When modelling multiple HFs over several time lags, the number of parameters to be estimated can approach or exceed the available observations, leading to unreliable estimation with traditional methods such as Ordinary Least Squares [17,18]. To overcome this high-dimensional regime, we employed a pipeline combining Multivariate Autoregressive (MVAR) models with LASSO regularization. LASSO promotes sparsity by automatically selecting only the most influential past states for predicting each HF’s future, yielding a robust and interpretable sparse directed network [19,20].

In particular, ATCOs were asked to perform realistic ATC simulations while their subjective, behavioural and neurophysiological data were collected. Since ATCOs’ performance is recognized to be impacted by multidimensional aspects such as mental workload, stress, emotions, attentional resource availability, attention, focus, and so on [21], we adopted the LASSO-regularized MVAR-cTE pipeline [16,22] to characterize the multivariate interactions among different HFs and to develop the neurophysiological data-driven HPE model. We have hence hypothesized that the HPE could be quantified as a dynamic function of directed predictive dependence between HFs, and that a more expansive, densely interconnected HF network characterized optimal performance states.

The manuscript is organized as follows: Section 2 describes the Materials and Methods, including the experimental group, ATM simulation protocol, behavioural and neurophysiological data collection, and the LASSO-regularized MVAR-cTE pipeline used to estimate directed HF interactions and derive the HPE model. Section 3 presents the results of behavioural, subjective and neurophysiological data, and the HPE characterization and correlation with individual ATCO performance. Section 4 discusses the theoretical and applied implications of the findings, acknowledges the study’s limitations, and outlines directions for future research. Section 5 draws the main conclusions of the proposed study.

## 2. Materials and Methods

**Figure 1:**
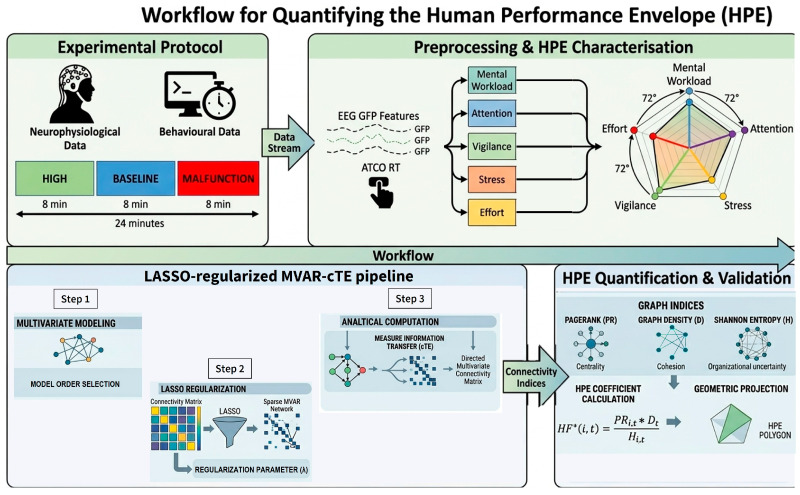
Schematic diagram of the experimental protocol, data collection and analysis, LASSO-regularized MVAR-cTE pipeline and HPE evaluation.

To better understand the different phases of the proposed study, Figure 1 shows a schematic overview of the experimental protocol, data collection and analysis, LASSO-regularized MVAR-cTE pipeline and HPE evaluation. These phases will be described in detail in the following sections.

### 2.1. Experimental Group

Twenty professional ATCOs from the École Nationale de l’Aviation Civile (ENAC, Toulouse, France) were involved in the study. The group consisted of 17 males and 3 females (mean age: 28 ± 12 years old). Three out of twenty controllers reported corrupted data due to technical issues; therefore, they were removed from the analyses. The experiments were conducted following the principles outlined in the Declaration of Helsinki of 1975, as revised in 2000. The experiments were approved by the Ethical Committee of ENAC (protocol code 2017/058). Informed consent was obtained from each controller on paper after the study explanation, and all the data were pseudonymized to prevent any association with controller identity in compliance with the current General Data Protection Regulation regulations.

### 2.2. ATM Scenario and Experimental Protocol

**Figure 2:**
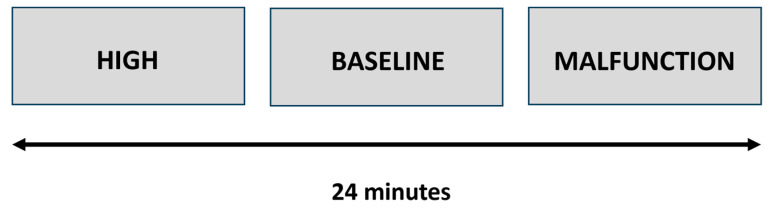
The ATM scenario consisted of three phases during which automation intervention changed, high automation (HIGH), low automation (BASELINE), and finally from low to high automation but with malfunctions (MALFUNCTION). Each phase lasted 8 min for a total duration of 24 min.

The ATC scenario consisted of three phases where the level of automation changed overtime from high automation (HIGH) to low automation (BASELINE), and finally from low to high automation but with malfunctions (MALFUNCTION). Each phase lasted 8 min for a total duration of 24 min (Figure 2).

**Figure 3:**
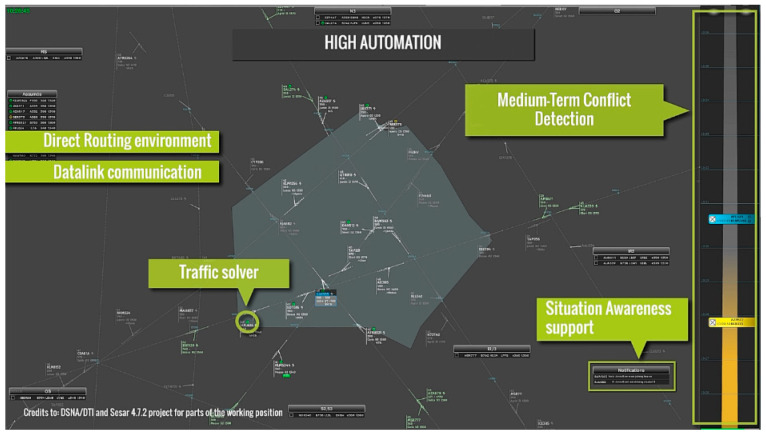
Overview of the ATM sector and summary of automation additional functionalities to the BASELINE condition on the RADAR interface.

The simulated sector (Figure 3) was an en-route airspace configuration used at ENAC for research and training; therefore, no specific familiarization on the radar platform and airspace had to be done before the experimentation. The traffic used was designed to be high-density all along the simulation. This means that the average number of aircraft at any time was almost stable and above fifteen. Furthermore, there could be up to three conflicts to solve at the same time. More details about the ATM sector can be found in [23].

### 2.3. Automation and Malfunction

Automation levels have been defined according to the SESAR level of automation taxonomy described in [24]. During the simulation, the BASELINE part was similar to what is provided in the current controller’s working positions and used in operations. In the HIGH parts of the scenario, specific tools were added to support decision-making, conflict handling, and improvements. In particular, the following automations were implemented in the HIGH condition:

- Conflict solver: It slightly modifies the speed of aircraft in conflict in order to increase the separation. The controller was informed of this action by a clock symbol in their track label.
- Situation awareness monitoring: This tool detects relevant information for ATCOs (i.e., aircraft compliancy to the planned 3D trajectory, communication status) and displays warning and alerts on the track label and a dedicated monitoring window.
- Conflict agenda: This tool shows in an agenda of incoming conflicts with detailed information within 15 NM.
- Highlighting of calling station: Whenever a pilot calls, the associated aircraft track label is highlighted on the radar screen.
- Reduce visual load: Specific information is filtered for non-relevant aircraft to reduce the visual load of the radar screen.
- Adapt STCA alert design: The short-term conflict alert design is enhanced in order to capture the attention of the air traffic controller.
- Controller pilot data link communication: Controller pilot data link communication allows for silent communication. It considerably helped to reduce ATCO workload linked to radio communication. The data link was activated in 60% of the traffic.

During the transition from HIGH to BASELINE, all those tools were removed from the controller’s working position interface. During the last phase (MALFUNCTION), the tools were back but inconsistencies appeared in the behaviour of the automation. In particular, the conflict solver flagged aircraft as being managed but the conflict was not solved at all, or the Medium-Term Conflict Detection showed incoherent incoming conflicts. These failures were the same for all ATCOs to avoid confounds in the experimental group.

### 2.4. Behavioural and Subjective Measure

To collect ATCOs’ behavioural data, a pop-up window appeared on the RADAR interface every minute allowing them to self-assess (instant self-assessment—ISA) and reflect on their own performance regularly. The presence of the pop-up enabled a more detailed and immediate analysis of the level of effectiveness of the actions taken in the last minute. In particular, ATCOs were asked to reply to the following question during the ATM scenario: “are you satisfied about how you are managing the traffic?”. The reaction times (ATCO RT) from the window pop-up onset and ratings (ATCO ISA) on a scale from 1 to 5 (i.e., 1: very low; 5: very high) have been collected. Reaction times provided valuable insights into how quickly and efficiently ATCOs processed the question and provided their ratings. Furthermore, a Subject Matter Expert (SME) closely observed the ATCOs and evaluated their overall performance in terms of safety, efficiency, and strategy. For each phase, SMEs assigned a rate (SME ISA), ranging from 1 to 5 (i.e., 1: very low; 5: very high). Since there are no standards for defining ATCO’s performance in terms of air traffic management, we evaluated ATCOs’ performance by combining the ISA scores and RTs. In this regard, the scientific literature demonstrated well that response speed (i.e., RT) is a direct index of the quality and efficiency of information processing in decision tasks [25,26]. As a consequence, we considered “1/RT” as a proxy for ATCOs’ cognitive readiness, hence high performance. The scientific literature also established the systematic self-assessment biases, that is, the tendency for operators to either over- or under-report their performance relative to objective standards [27,28]. The integration of these perspectives provided us with a comprehensive overview of controller performance, enabling us to evaluate not only cognitive readiness but also subjective perception and critical analysis from industry experts. The Performance Index was therefore defined by the following formula:

(1) $$Performance\;Index\;=\;\left(\frac{SME\;ISA}{ATCO\;ISA}\right)\;*\frac{1}{ATCO\;RT}\;$$

The rationale behind Equation (1) is that the SME’s evaluations were the “gold standard” for performance evaluation. In particular, when SME ISAs were higher than ATCO ISAs, the ratios, hence weights of RTs, were >1, which meant that the performance was good. On the contrary, when SME ISAs were lower than ATCO ISAs, the weights were <1 because it meant that SME evaluated the ATCOs’ performance as poor, and the corresponding RTs were penalized accordingly. In other words, the normalization adopted in our study aimed to adjust for the biases mentioned previously and provide an accurate estimation of ATCOs’ performance while dealing with air traffic control activity.

### 2.5. Neurophysiological Data Recording and Analysis

ATCOs’ brain activity, their electroencephalographic (EEG) data, was recorded using the BEmicro (EBNeuro, Florence, Italy) digital monitoring system with a sampling frequency of 256 Hz. Sixteen Ag/AgCl gel-based electrodes were positioned over the ATCO’s scalp according to the 10–20 International System [29]. Specifically, electrodes were placed at *Fpz*, *F3*, *Fz*, *F4*, *AF3*, *AFz*, *AF4*, *C3*, *C4*, *P3*, *Pz*, *P4*, *POz*, *O1*, *Oz*, and *O2*, referenced to both the mastoids and grounded to the *Cz*. Prior to data collection, electrode impedance was verified to be below 10 kΩ to ensure adequate signal quality [30]. Before analysis, the EEG data underwent a pre-processing phase aimed at identifying and correcting physiological and non-physiological artefacts unrelated to cerebral activity of interest (e.g., ocular movements, muscle activity, body motion). Signals were band-pass filtered between 2 and 30 Hz using a 5th-order Butterworth filter. Eye-blink artefacts were detected and corrected using the o-CLEAN method [31,32]. Additional artefacts, including those from muscular activity and movements, were identified and removed using ad hoc algorithms based on the EEGLAB toolbox [33]. The data reported an average of 20% artefact epochs [31,32]. From the artefact-free EEG data, the global field power (GFP) was computed within the Theta, Alpha, Beta and Gamma bands which were individually defined based on each controller’s Individual Alpha Frequency [34]. The Individual Alpha Frequency was determined from a 60 s baseline acquired with controllers’ eyes closed (a condition that reliably enhances the Alpha peak). Subsequently, EEG GFP features were extracted for each 1 s epoch using a Hanning window of matching length, yielding to a frequency resolution of 1 Hz. On the basis of the original definition of the HPE proposed by Bittner [4], we targeted five HFs to provide an accurate neurophysiological characterization of the HPE: mental workload, attention, stress, vigilance and effort [5,6,13]. The corresponding neurometrics were derived in accordance with previous works [35,36,37,38,39,40,41,42,43,44]:

Mental Workload = Theta frontal/Alpha high parietal(2)

Attention = −Alpha frontal(3)

Vigilance = −Beta frontal(4)

Stress = Beta High parietal(5)

Effort = Theta frontal(6)

Each neurometric consisted in a vector of L = 3 (phases) * 8 (duration of each phase in minutes) * 60 (resolution of a second) = 1440 samples. Since each neurometric was a ratio or linear combination of specific EEG bands, missing values (NaN) arose at any time point corresponding to artefact rejections. Artefact rejection therefore produced gaps in each neurometric series independently. For example, a NaN epoch in the frontal Theta band produced a NaN in mental workload and effort but left the stress and vigilance unaffected. Similarly, NaN values could be found in frontal Theta and parietal band for the mental workload but in different time points. As a consequence, to obtain a gap-free input matrix for MVAR parameter estimation (which requires a non-singular, fully observed multivariate time series [45,46,47]), missing neurometric values (NaNs) were estimated using a procedure based on the *synthetic minority over-sampling technique* [48] applied independently to each of the five HF time series. In particular, when NaNs were found in a given neurometric time series, the nearest neighbours (K = 5) were identified among the non-missing observations of that same series, and synthetic values were generated by linear interpolation of neighbours’ values [48]. This step was necessary to ensure temporal alignment across the five HF series prior to MVAR estimation [45]. Finally, as the LASSO-regularized MVAR-cTE model requires that all input series have comparable numerical scale to ensure equal treatment of processes under the L1 penalty [17,18], and the neurometrics were standardized using z-score transformation.

### 2.6. LASSO-Regularized MVAR-cTE Pipeline

The definition of the neurophysiological-based model of the HPE started from its original formulation [4] and required a methodological approach capable of (i) estimating directed functional connectivity among all HFs simultaneously, (ii) maintaining estimation reliability despite the high dimensionality of the MVAR model relative to the available observations (1440 samples, 5 HFs), and (iii) quantifying these interactions through a multivariate information-theoretic measure. In the context of this study, the terms ‘causal’ and ‘interactions’ are employed strictly in the information-theoretic and Granger-predictive sense. These terms refer to the directed predictive dependence identified between the HF time series by the MVAR model, rather than implying underlying physical or biological causal mechanisms [49]. Notably, traditional linear methods for assessing directed connectivity, such as Granger Causality (GC) and its multivariate extensions (multivariate Granger causality) operate on a principle of temporal precedence and predictability: if the past of time series *X* improves the prediction of the future of time series *Y* beyond the past of *Y* itself, a directed influence from *X* to *Y* is inferred [50]. Also, GC-based models are inherently bivariate or low-order multivariate, struggling to differentiate between a true direct connection and an indirect interaction mediated by a third or more processes (e.g., 5 HFs). To address this limitation, the present study adopted the LASSO-regularized MVAR-cTE approach implemented in the theoretical example code publicly available in the PID-LASSO toolbox by Antonacci et al. [17,18] (https://github.com/YuriAntonacci/PID-LASSO-toolbox (accessed on 29 June 2023)). Specifically, only the LASSO-regularized MVAR model identification and the subsequent analytical cTE computation were employed from this toolbox; the Partial Information Decomposition (PID) components of the toolbox were not used in the present study.

LASSO is a regularization technique that imposes an L_1_ penalty on the model coefficients, promoting sparsity and yielding compact model representations particularly suited to high-dimensional settings where the number of parameters risks exceeding the available observations, as is the case in linear VAR or state-space model identification. The core innovation lies in using LASSO not merely as a filter, but as an integral part of the connectivity process. In other words, we employed LASSO to automatically identify the sparse set of the most influential past states from all possible HFs for predicting each HF’s future. The resulting sparse MVAR model inherently represented a directed functional network, filtering out weak or spurious connections (i.e., interactions). This sparse network is then used to analytically compute the multivariate conditional transfer entropy (cTE) along each identified connection, yielding a stable and interpretable measure of directed information flow [17,18]. The analysis pipeline for the HPE model definition consisted therefore in three methodological steps:

Step 1—Multivariate linear model specification. A vector autoregressive (VAR) model of order *p* was specified for the five HFs’ state vector $X\left(t\right)={\left[Mental\;Workload\left(t\right),Attention\left(t\right),Stress\left(t\right),Vigilance\left(t\right),Effort\left(t\right)\right]}^{T}\in R^{N\times 1}$ with N=5.

(7) $$X\left(t\right)=\sum_{k=1}^{p}A_{k}X\left(t-k\right)+W\left(t\right)$$

where $A_{k}\in R^{N\times N}$ are the coefficient matrices, and W(t) $\in R^{N\times 1}$ is the vector of white noise innovations with covariance matrix $\Sigma =E\left[W\left(t\right)W{\left(t\right)}^{T}\right]\;\in R^{N\times N}$. The model order *p* was selected per each ATCO using the Bayesian Information Criterion (BIC) and Akaike Information Criterion (AIC); see Section 2.7 for more details, over the range *p* ∈ {1, …, 20}. The VAR model provided the linear dynamical system from which all subsequent information-theoretic quantities were derived analytically, without requiring the estimation of multivariate probability densities directly from the data [45,49].

Step 2—Sparse model identification via LASSO. Ordinary least squares (OLS) estimation of the VAR coefficient matrices was ill-suited to our dataset. Specifically, considering *N* = 5 processes and *p* lags ranging from 1 to 20, the parameter matrix had T = (*N* * *N* * *p*) = (5 * 5 * *p*) = 25 * *p* autoregressive parameters. With L = 1440 observations and controller-specific *p* orders, the observation-to-parameter ratio (L/T) ranged from 14.4 (*p* = 4; controllers S7, S14) to 5.2 (*p* = 11; controllers S8, S12). Twelve of the 17 controllers had L/T < 10 which placed the estimation problem in the data-paucity regime, for which Antonacci et al. [17,18] demonstrated that LASSO outperformed OLS in both network topology recovery and conditional transfer entropy (cTE) estimation accuracy. To cast the VAR model identification as a regression problem, the lagged observations of all processes are stacked into the regressor vector $Z\left(t-1\right)={\left[X{\left(t-1\right)}^{T},X{\left(t-2\right)}^{T},\;\ldots ,X{\left(t-p\right)}^{T}\right]}^{T}\in R^{Np\times 1}$. Under this definition, each scalar equation of the VAR model can be written as $X_{j}\left(t\right)=Z{\left(t-1\right)}^{T}\beta_{j}+W_{j}\left(t\right)$, where the coefficient vector $\beta_{j}\in R^{Np\times 1}$ corresponds to the j-th row of the stacked VAR coefficient matrix [$A_{1},A_{2},\ldots ,A_{p}$] $\in R^{N\times Np}$, collecting all autoregressive coefficients for predicting the j-th process across all lags and all source processes. LASSO was therefore applied to identify each row of the VAR coefficient matrix [17,18] by solving the following penalized regression problem:

(8) $${\hat{\beta }}_{j}=\arg\underset{\beta_{j}}{\min}\left(\sum_{t=p+1}^{L}{\left(X_{j}\left(t\right)-{Z\left(t-1\right)}^{T}\beta_{j}\right)}^{2}+\lambda_{j}|{|\beta }_{j}{\left|\right|}_{1}\right)$$

where the summation runs over all *L − p* available samples, with *L* being the total recording length and *p* the model order; $Z\left(t-1\right)\in R^{Np\times 1}$ is the regressor vector of stacked lagged observations as defined above, $\beta_{j}\in R^{Np\times 1}$ contains the VAR coefficients for the j-th process, $\lambda_{j}$ is the regularization parameter selected via cross-validation to minimize the prediction error, and the $L_{1}$ penalty term $||\beta_{j}|{|}_{1}$ promotes sparsity by driving non-significant coefficients to exactly zero [19,20]. Once LASSO has been solved for all j = 1, …, N, the full set of VAR coefficient matrices $A_{1},\ldots ,A_{p}$ is recovered by partitioning the matrix ${\left[\beta_{1},\beta_{2},\ldots ,\beta_{N}\right]}^{T}\in R^{N\times Np}$ into *p* consecutive blocks of N columns each, yielding $A_{k}\in R^{N\times N}$ for k = 1, …, *p*. The sparse VAR model so identified is then passed to Step 3 for the analytical computation of cTE. The L_1_ penalty drove many of the 25 *p* VAR coefficients to exactly zero, reducing the effective parameter count below T, substantially improving estimation reliability, and yielding a sparse directed network of HF connections [17,18]. Crucially, in our study, LASSO was employed as a model identification tool and not as an information-theoretic method.

Step 3—Analytical computation of the cTE from the state—space (SS) model. The sparse VAR model identified in Step 2 was converted to its equivalent SS representation, following the approach of Barnett & Seth [49] and Faes et al. [51]. This conversion allows for the analytical computation of information-theoretic measures directly from the model parameters, avoiding the biases associated with direct probability density estimation from finite samples. As established by [49], under the linear Gaussian assumption cTE is equivalent to conditional GC. For each directed pair $\left(X_{i}\to X_{j}\right)$, the cTE was computed by conditioning on the full past of the remaining N−2 = 3 HFs, here denoted by the conditioning set $S=X_{k}:k\neq i,k\neq j$, representing the joint past of all processes except source i and target j. This multivariate conditioning ensured that the estimated information flow reflected a direct predictive link. Specifically, the residual variances of the unrestricted ($\sigma_{\left\{u\left(j|S\right)\right\}}^{2}$) and restricted ($\sigma_{\left\{r(j|S\backslash \{i\})\right\}}^{2}$) models were computed analytically via the discrete algebraic Riccati equation [49,52] without refitting separate models. Following [17,18], the cTE is defined as

(9) $$cTE\{i\to j\;|\;S\}=\frac{1}{2}*ln\left(\frac{\sigma_{\left\{r(j|S\backslash \{i\})\right\}}^{2}}{\sigma_{\left\{u\left(j|S\right)\right\}}^{2}}\right)\;$$

where $\sigma_{\left\{u\left(j|S\right)\right\}}^{2}$ is the residual variance of the model predicting the future of $X_{j}$ from the past of $X_{i},\;X_{j},\;\mathrm{and}\;S$, while $\sigma_{\left\{r(j|S\backslash \{i\})\right\}}^{2}$ is the residual variance of the restricted model that excludes the past of $X_{i}$; and S denotes the joint past of all HFs processes except *i* and *j*. The non-negativity of cTE is guaranteed by the property $\left(\sigma_{u}^{2}\;\leq \sigma_{r}^{2}\right)$, with cTE = 0 when *X*_i_ carries no additional predictive information about *X*_j_ beyond the conditioning set. Because conditioning was performed over the full complement set *S* (all remaining HFs simultaneously, N − 2 = 3), cTE constituted a genuinely multivariate measure of directed information flow [17,51,53]. To obtain a single node-level scalar cTE(*i*), the multivariate cTE was computed for each target *j ≠ i* and averaged across all N − 1 possible targets by the following equation:

(10) $$cTE\left(i\right)=\left(\frac{1}{\left(N-1\right)}\right)*\sum_{\{j\;\neq \;i\}\;}{cTE}_{\left\{i\to j\;|\;S\right\}}\;$$

This scalar summarized the total directed informational output of HF_i_ across the entire network, controlling for the network-wide context at each time point. A high cTE(i) therefore indicated that the past of HF_i_ consistently provided significant predictive information about the futures of all other HFs even after accounting for the full network context (in our study, this assumption was associated with performance degradation). Conversely, a low cTE(i) indicated that HF_i_’s dynamics were already well-explained by the rest of the network, suggesting a more integrated, cooperative information structure.

Importantly, as described in Section 2.9, the node-level scalar cTE(*i*) was not directly used in the HPE coefficient formula; rather, the cTE connectivity matrix served as the basis for computing the graph-level indexes (PageRank, Graph Density, and Shannon Entropy) that enter the HPE model.

### 2.7. MVAR Model Order Selection

The selection of optimal model order for MVAR models typically employs information-theoretic criteria, predominantly the Akaike Information Criterion (AIC) [54] and the Bayesian Information Criterion (BIC) [55], which balance model goodness-of-fit against parametric complexity. In particular, in our study, MVAR model order *p* was selected independently for each ATCO by evaluating the AIC and BIC over a range of *p* ∈ {1, …, 20}. When AIC and BIC disagreed, BIC was preferred as its stronger logarithmic complexity penalty yields a consistent estimator that converges to the true model order under the correct model class, and it controls false-positive connection detection more conservatively in the large-sample limit [56,57]. This is also advantageous for controlling model complexity relative to the available sample size, in real and noisy data [58,59], producing a more stable and interpretable model, and is often considered safer when the goal is to make inferences about the existence of connections (e.g., in network analysis).

### 2.8. LASSO Regularization Parameter λ Selection

The LASSO regularization parameter *λ* controls the sparsity of the estimated MVAR coefficient matrices and it was the single most consequential free parameter in the analysis pipeline as it determined which directed HF connections were retained as non-zero, and thereby defining the sparse network from which Graph’s Density (D), PageRank (PR) and Shannon’s entropy (H) values were derived [19,20]. In general, too large λ values yield an empty network, while too small λ values retain spurious connections driven by noise. The λ was therefore selected independently for each ATCO via the k-fold cross-validation approach [60,61]. In particular, controllers’ HF time series were partitioned into k = 10 sequential, non-overlapping folds of equal length (1440/10 = 144 samples). The cross-validation process was performed iteratively: in each round, the model was trained using *k −* 1 subsets (training set), and its performance was then validated on the single remaining held-out subset (validation set). This procedure was repeated *k* times, with each of the 10 folds serving as the validation set exactly once. A vector of λ log-spaced values (ranging from 10^−5^ to 1000) was evaluated considering as a validation criterion the mean squared error of the prediction MVAR models. For each ATCO, the λ value minimizing the mean squared error was finally retained as a regularization parameter.

### 2.9. Human Performance Envelope (HPE) Characterization

Graph indexes are capable of objectively describing the network derived from the application of the LASSO-regularized MVAR-cTE pipeline to the HFs data series [62,63,64,65]. These indexes synthesize the complex dynamics of HF relationships over time, providing a concise yet comprehensive representation of the variation and structure of connections between HFs. The goal was therefore to accurately and fully capture the interactions between different HFs, allowing for a deeper understanding of underlying processes and their implications for behaviour and cognitive functions. For the HPE model characterization, we considered the PageRank, Graph Density Index, and Shannon Entropy. The reason behind this selection is described here below:

- PageRank (PR) is used to evaluate the importance of each node based on the number of incoming relationships and the rank of the related source nodes [65]. PR effectively returns the probability distribution representing the likelihood of visiting a particular node (i.e., HF) through a random traversal of the graph. Essentially, the PR assesses the importance of each HF based on its connectivity with other high-ranking nodes (i.e., HFs) in the graph. This means that nodes with a large number of inbound links from high-ranking nodes tend to have a higher PR score. The resulting probability distribution allows for understanding which nodes are more central or influential in the overall structure of the network. The PR of the *i*-th node (e.g., HF) was calculated by the following formula
  (11) $$PR\left(i\right)=\frac{1-d}{N}+d\sum_{j\in M(i)}\frac{PR(j)}{C(j)}\;$$
  where *d* (damping factor) was set to 0.85, *N* is the total number of nodes (e.g., HFs = 5), *M*(*i*) the set of nodes *j* that has directed, functional connections pointing to node i, *PR(j)* the current PageRank score of the neighbouring node j that is connected to node i, and *C*(*j*) the total number of outgoing connections from node j to other brain regions. The factor “PR(*j*)/*C*(*j*)” represents the share of node *j*’s influence that flows to node *i*, and it can assume values within the range [0 ÷ 1].
- Graph Density (D) is a measure that indicates how many connections between nodes exist compared to how many connections between nodes are possible [66]. Since MVAR-cTE estimates a directed functional graph, for a directed graph with N vertices and *E* edges, the maximum number of possible edges is N(N − 1) and the density D is calculated as:
  (12) $$D=\frac{\left|E\right|}{\left|N|(|N|-1\right)}$$
  The density of a graph (D) hence provides an indication of the strength of connections within the network. It represents the ratio between the actual number of connections present in the graph and the maximum number of connections that could exist considering all possible links between the nodes in the network. As a consequence, it can assume values within the range [0 ÷ 1]. A high D suggests a densely interconnected network, where most nodes are connected to each other. Conversely, a low D indicates a more dispersed or fragmented network, with few connections between nodes. Measuring the D of a graph allows for understanding the cohesion and complexity of relationships within the network, providing valuable information about its structure and its resilience to variations and external events.
- Shannon’s entropy (*H*) is essentially a measure of the uncertainty or information contained in a random data source. More precisely, it is the average amount of information produced by a stochastic data source [67,68]. When discussing “information,” it refers to how significant or surprising a new value in a variable is. If Shannon Entropy is high, it indicates that there are many different possibilities or a lot of variation in the data, so each new value obtained provides a considerable amount of additional information. Conversely, if entropy is low, it implies there are few possibilities or little variation, thus each new value does not contribute significantly to the information. To operationalize this for the HPE model, the directed cTE connectivity matrix (identified in Section 2.6) was first converted into a binary adjacency matrix ($M_{bin}$) where 1 indicates a significant causal link and 0 indicates its absence. We then computed the Shannon Entropy of this binary pattern using a binning estimation method
  (13) $$H\left(i,t\right)=-\sum P\left(x_{i}\right)\log P\left(x_{i}\right)$$
  where $P\left(x_{i}\right)$ represents the probability distribution of the binary connectivity patterns. A high H indicates a fragmented or disorganized connection pattern, suggesting a degradation in the controller’s integrated mental state. This captures the organizational “uncertainty” of the directed dependencies between HFs.

Based on the above, we hypothesized that each HF’s contribution to the HPE at time *t*, denoted HF*(i, t), can be characterized by a composite coefficient derived from the product of its node-level centrality PR(i, t) and the global network cohesion D(t), divided by the organizational uncertainty H(i, t) of the connectivity pattern:

(14) $$HF*(i,\;t)=\left(\frac{PR\left(i,t\right)*D(t)}{H\left(i,t\right)}\right)$$

Interpretations of Equation (14) include the following one. PR(i, t) captures the local influence of HF*i* within the directed network at time *t*: a HF that receives many connections from other influential HFs contributes more to the overall performance state. D(t) acts as a global amplifier: a densely interconnected network amplifies the contribution of each HF to the HPE polygon, while a fragmented network attenuates it, consistently with the HPE theoretical model [4,6] that individual HF states are meaningful only in the context of the overall network structure. H(i, t) acts as a penalty term: as the organizational uncertainty of the connectivity pattern increases, reflecting a more disorganized and less coordinated HF interaction structure, the contribution of each HF to the HPE is proportionally reduced. The combined effect of these three indexes ensures that HF*(i, t) is high only when a HF is simultaneously central (high PR), embedded in a dense network (high D), and operating within a well-organized connectivity pattern (low H).

**Figure 4:**
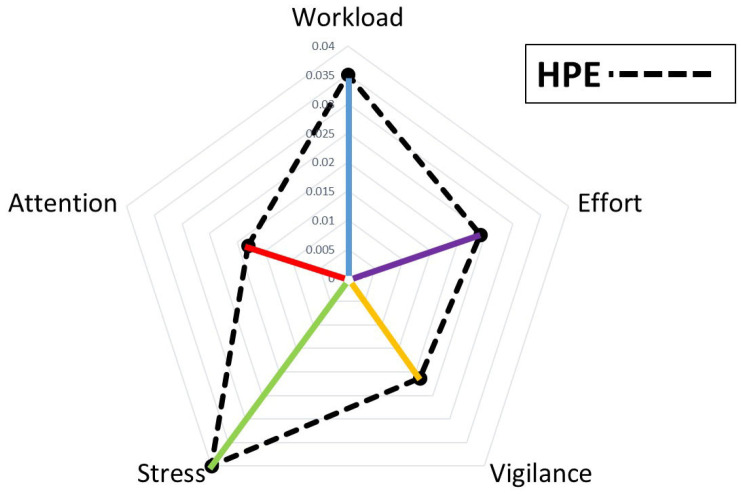
Graphical representation of the HPE model characterization at a given time t. Each HF can be modelled by a coefficient calculated through the combination of its PageRank, Graph Density and Shannon Entropy. These coefficients (coloured lines) define a vertex of a polygon. The area of such a polygon is the HPE measure at a given time t (dashed lines).

This formulation is consistent with the brain connectivity literature that combines global and local network metrics within composite indexes, on the grounds that a node’s functional contribution depends not only on its local properties but also on the global network context in which it operates [69,70,71]. The resultant HF* values for the five HFs defined points in a 5-dimensional performance space ranging from 0 to 1. For visualization and scalar quantification, we projected them onto a 2D space, where the area enclosed by the vertices HF* provided a single and integrative metric of the HPE at time *t* (Figure 4). This follows the geometric intuition that a larger area of the polygon (an irregular pentagon) represented a greater interaction across all HFs, that is, a higher HPE [4,6,13]. The measure of the controllers’ HPE was therefore obtained by measuring the area of such an irregular pentagon by using the Gauss’s Area Formula (or Shoelace Formula) as described in [72,73]:

(15) HPE(t)≜Polygon Area (t)

A graphical representation of the HPE model characterization is reported in Figure 4. By analyzing the area of the polygon, which reflects HF interaction and intersection, a more complete and accurate view of the ATCO’s performance can be obtained. To ensure that the polygon area was comparable across all subjects and conditions, the five HFs were assigned to fixed and equally spaced angular positions of 72° (360°/5 HFs = 72°) that were kept constant across all ATCOs, time points, and conditions. The Shoelace Formula was therefore applied to the resulting coordinates in this fixed angular order allowing direct comparisons.

## 3. Results

### 3.1. Behavioural Results

The ATCOs’ Performance Index has been analyzed across the three experimental conditions. Although a slight decrease has been noted in the BASELINE condition with respect to the ones where the automation was enabled, the repeated measure ANOVA did not report any statistical effects (F = 2.02; *p* = 0.15; ƞ2 = 0.096).

### 3.2. ATCO ISA Results

The repeated measure ANOVA on the ISA scores provided by the controllers during the execution of the ATM scenario did not report any statistical differences (F = 0.67; *p* = 0.52; ƞ2 = 0.04). In particular, the controllers exhibited almost the same perception under the different experimental conditions.

### 3.3. SME ISA Results

Similarly to the controllers, the statistics of the ISA scores provided by the SMEs along the ATM scenario did not report any significant effects (F = 2.4, *p* = 0.1, ƞ2 = 0.11).

### 3.4. MVAR Model Order Selection

Table 1 reports the model order *p* selected for each ATCO.

**Table 1:** Model order (*p*) selected for each ATCO.

Controller ID	Model Order (p)
S1	7
S2	5
S3	8
S4	9
S5	9
S6	7
S7	4
S8	11
S9	9
S10	5
S11	7
S12	11
S13	7
S14	4
S15	5
S16	10
S17	6

### 3.5. HPE Results

HPE values of ATCOs have been averaged within each of the three experimental conditions (HIGH, BASELINE and MALFUNCTION). The repeated measure ANOVA on these HPEs did not return any significant effects (F = 1.15, *p* = 0.33, ƞ^2^ = 0.067) along the ATM scenario.

### 3.6. HPE Corresponding to High and Low Performance

**Figure 5:**
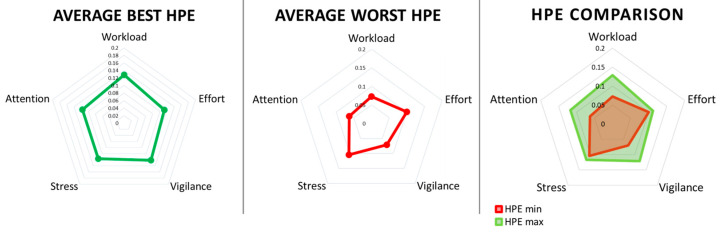
Representation of the average HPE during the best (HPE max, green colour) and worst (HPE min, red colour) performance.

Based on the original HPE definition, the ATCOs’ HPE should have changed with their performance levels. In this regard, we identified the temporal moments *t* corresponding to controllers’ maximum (best) and minimum (worst) performance values. In particular, 42% of the worst performance occurred during HIGH, 42% during BASE and 16% in MALFUNCTION. The best performance occurred for 50% of cases in HIGH, 22% in MALFUNCTION, and 28% in BASE. In line with these moments, we computed the corresponding HPE values and then performed statistical analysis on these distributions. Figure 5 shows the average HPE during the best (HPE max, green colour) and worst (HPE min, red colour) performance.

**Figure 6:**
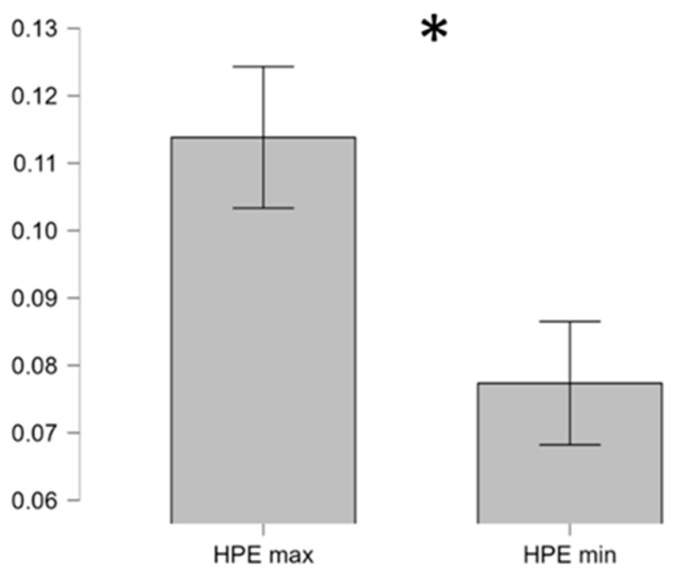
The two-tail paired *t*-test on the average HPE showed a significant difference between the best (HPE max, green polygon in Figure 5) and worst (HPE min, red polygon in Figure 5) performance condition. Error bars represent the standard error of the mean across controllers. Asterisk symbol “*” indicates statistical significance (*p* < 0.05).

In particular, it is possible to note how the area of the corresponding polygon during the worst performance (HPE min = 0.07) was lower than the one during the best performance (HPE max = 0.11). As reported in the HPE model definition [4,6,13], a bigger area for the HPE suggests more effective performance, indicating that the controllers operated successfully across a wider range of cognitive conditions. Conversely, a smaller area for the HPE suggests more limited performance, indicating that the controllers may have encountered greater difficulty. The paired two-tail *t*-test on average HPE values between the best and worst conditions showed a significant difference (t = 3.06, *p* = 0.009, Cohen’s d = 0.82), suggesting HPEs changed significantly when the controllers performed better; see Figure 6.

**Figure 7:**
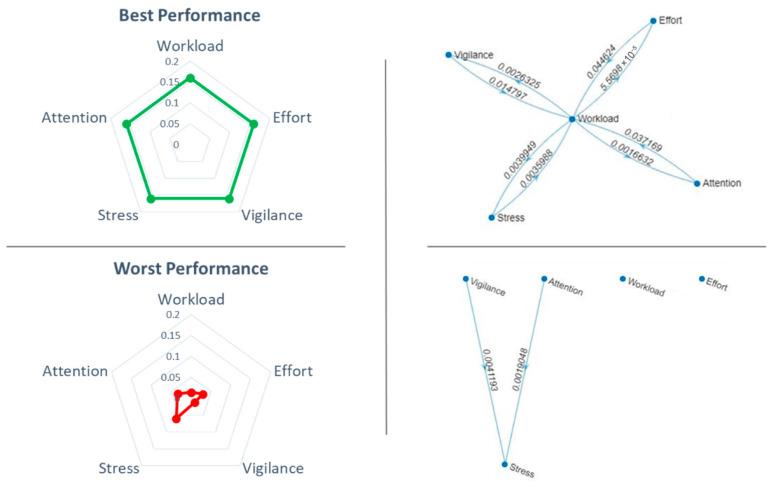
Number of connections among HFs of a representative controller corresponding to the best (green line) and worst (red line) performance conditions.

It was also very interesting to investigate what happened in terms of HF interactions (i.e., number of links) under these conditions. We found that when the ATCOs’ performance was the best, all the considered HFs (i.e., mental workload, effort, attention, stress and vigilance) were densely interconnected (top panel, right side of Figure 7). On the contrary, when the controllers’ performance assumed the worst value, most of the links among the considered HFs drastically reduced (bottom panel, right side of Figure 7). In the left side of Figure 7 the HPEs during, respectively, the best and worst performance of a representative controller are represented.

**Figure 8:**
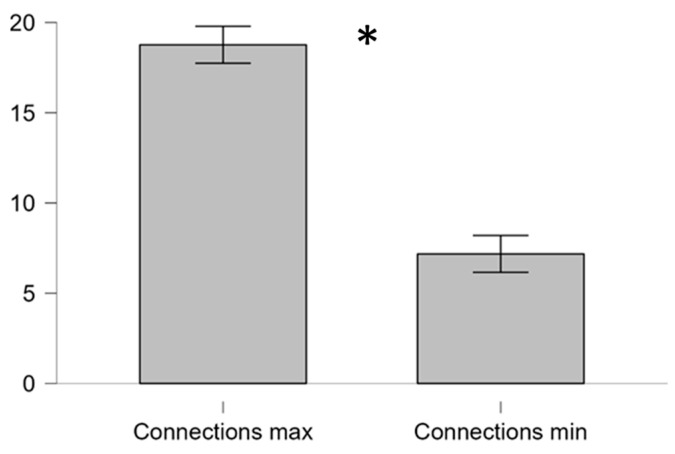
The two-tail paired *t*-test on the average number of connections related to the best (connection max) and worst (connection min) performance showed a significant difference. Error bars represent the standard error of the mean across ATCOs. Asterisk symbol “*” indicates statistical significance (*p* < 0.05).

In particular, the paired two-tail *t*-test on the average number of connections among HFs related to the best and worst performance condition showed a significant difference (t = 8.02, *p* < 0.001, Cohen’s d = 1.95), suggesting that interactions and dynamics among HFs, hence the HPE, changed significantly when the ATCOs performed better (Figure 8).

### 3.7. Correlation Between HPE and Performance Index

**Figure 9:**
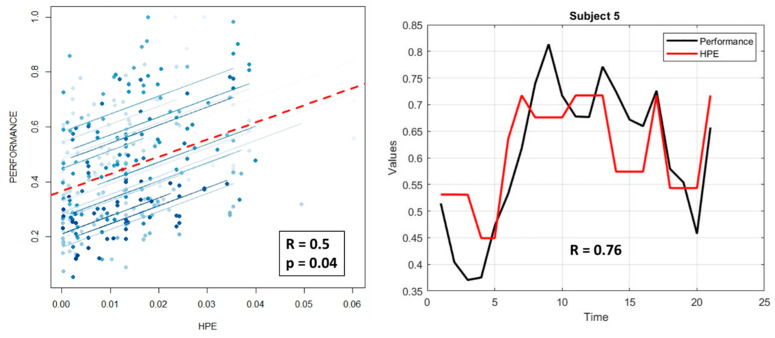
The repeated measure correlation (rmcorr) analysis on controllers’ performance and the corresponding HPE values over time returned a positive (R = 0.5) and significant (*p* = 0.04) correlation (left panel). The blue lines represent the correlation trend of each ATCO, while the dashed red line reports the trend of the group correlation. In the right panel it is possible to note the strong positive correlation (R = 0.76) for a representative ATCO.

To better evaluate the proposed neurophysiological-based HPE model, we investigated the relationship between individual performance and the corresponding HPE values over time. In this regard, repeated measure correlation (rmcorr) analysis [74] was performed on these parameters to obtain a validation at both single-ATCO and experimental group levels. For each ATCO, HPE values were averaged within each minute to have vectors of the same length as the performance ones. Figure 9 shows the results of the rmcorr analysis reporting a positive overall correlation coefficient R = 0.5 and a significant statistic with *p* = 0.04 (left panel). The correlation between the HPE and performance of a representative ATCO (*S5*) is reported in the right panel of Figure 9. It is possible to note the strong positive correlation (R = 0.76) between the two parameters.

## 4. Discussion

The objective of the work was to develop a neurophysiological-based model of the human performance envelope (HPE). The HFs considered for the HPE characterization were the mental workload, vigilance, stress, effort and attention. These HFs allowed for a comprehensive understanding of the ATCOs’ cognitive and emotional states. However, the focus was not solely on the HFs’ intrinsic values to define HPE, but rather on exploring their mutual influence and interaction. The objective was therefore to find a model that, considering the combination and interaction among the different HFs, could accurately describe the ATCOs’ performance. In doing so, we had to understand how these HFs integrated and influenced each other. In this regard, the LASSO-regularized MVAR-cTE model was adopted. From the connectivity networks returned by the pipeline, we identified three graph indexes able to describe the interaction between HFs: PageRank (PR), Graph Density (D) and Shannon Entropy (H). The PR and D take into account the degree and number of relationships of each HF with the others. At the same time, when the H increases, indicating more disorder and complications in the subgraph, performance decreases. Consequently, the contribution of each HF to the controller’s HPE was calculated using Equation (14). Such values defined a polygon whose area was the proposed HPE measure through Equation (15).

The analyses on behavioural, subjective and neurophysiological data across the three experimental phases (HIGH, BASELINE, MALFUNCTION) did not reveal any significant difference. This result could be explained by the fact that ATCOs always strive to perform at their best regardless of the ongoing situation.

However, based on the original HPE definition, significant differences were found in to correspond with conditions of highest (best) and lowest (worst) performance. In particular, both the area of polygons charactering the average HPE (Figure 6) and average number of connections (i.e., links) among HFs (Figure 8) under such conditions reported significant differences. In other words, operational resilience and performance were underpinned by a densely interconnected operators HFs, supporting the directed and multivariate model of the HPE.

Furthermore, to assess the potential relationship between the proposed neurophysiological HPE characterization model and human performance, repeated measure correlation analysis was performed on HPEs and the corresponding ATCO performance over time. The result returned positive (R = 0.5) and significant (*p* = 0.04) evidence of how high HPE values corresponded to high performance, and vice versa (Figure 9).

It should be noted that the present study is intended as a first proof-of-concept of a neurophysiological framework for HPE quantification. The analysis pipeline involves multiple sequential modelling steps (i.e., model order selection, LASSO regularization, VAR-to-state-space conversion, and analytical cTE computation), and while each step relies on well-validated procedures, the observed correlations between HPE metrics and performance conditions should be interpreted as exploratory evidence of convergent validity rather than as a mechanistic demonstration of causality. Replication in larger, independent cohorts with pre-registered confirmatory analyses is required before drawing definitive conclusions.

Although the results were very promising for understanding the relationships between a multivariate combination of neurophysiological indicators and ATCOs’ performance, we have to highlight some limitations and actions for the next study. In particular, the following should be noted:

- Missing neurometric values (NaNs) arising from artefact-rejected epochs were substituted using the synthetic minority over-sampling technique. This procedure could have introduced mild smoothing at imputed positions in HFs. Given the slow timescale of cognitive state dynamics (i.e., HFs), this effect was expected to be small, but it should be formally validated in future works.
- Controller performance assessments were derived from subjective ratings (SME and ATCO); therefore, they might not fully represent their operational performance in managing air traffic.
- The sample size consisted of twenty controllers; therefore, an enlarged group is needed to further validate the result. Additionally, validations in other contexts (e.g., automotive, surgery) will be considered.
- Other MVAR models will be considered to take into account non-linear connections among HFs (e.g., modification of the backward-in-time selection, and Partial Multivariate Information-based Non-uniform Embedding) for the HPE characterization.
- More HFs (e.g., engagement, mental fatigue) and neurophysiological parameters like heart rate, skin conductance, and eye tracking will be integrated into the HPE model definition for a more comprehensive and accurate understanding of dynamics for HPE characterization.
- The strategy of selecting extreme performance moments (best and worst) and then comparing corresponding HPE values using a paired *t*-test was susceptible to regression-to-the-mean effects. However, we wanted to test the original HPE theoretical model, that is, that the HPE polygon area is significantly larger at moments of peak performance than at moments of performance degradation. Future works performed in laboratory settings and controlled experimental conditions will allow for better investigation into this aspect over time and not only at specific time moments.

## 5. Conclusions

Understanding the HPE is essential for optimizing performance and safety in high-stakes environments. By considering behavioural and physiological factors, and leveraging advanced monitoring technologies, we can ensure that individuals operate within their performance limits, thereby reducing the risk of errors and accidents. A model of the HPE can guide the development of advanced competency-based training, pushing trainees to safely explore their boundaries in simulation contexts. Indeed, by making the invisible boundaries of human performance visible, measurable and central to design, we can provide a more efficient and capacious, and above all, more resilient and human-centric training framework, ensuring that humans remain the keystone of safety in increasingly complex working contexts. Nonetheless, given the exploratory nature of the present findings and the relatively small sample size, these conclusions should be considered as directional rather than definitive, and future confirmatory studies are warranted.

This work does not simply mark the end point in the research on HPE, but rather represents a significant step towards achieving something concrete. The goal is to provide a tangible contribution that can serve as inspiration for future studies and lead to necessary improvements to optimize the HPE to its full potential.

Additionally, developing integrated models that combine physiological, behavioural, psychological, and environmental data provide a more holistic understanding of the HPE. These models can predict performance outcomes under various conditions and help in designing better support and adaptive systems [75]. These adaptive systems can respond to real-time monitoring data and adjust task demands or provide support accordingly to help keep individuals’ performance within their HPE. These systems can enhance performance and safety in high-stakes environments [76].

## Data Availability

The dataset presented in this article is not available because raw data could be accessed only by the Scientific Officer and Assistant Researchers as reported in the Informed Consent.

## Abbreviations

The following abbreviations are used in this manuscript:

AIC	Akaike Information Criterion
ATCO	Air Traffic Controller
ATM	Air Traffic Management
BIC	Bayesian Information Criterion
cTE	Conditional Transfer Entropy
D	Graph Density
EEG	Electroencephalography
ENAC	École Nationale de l’Aviation Civile
GC	Granger Causality
GFP	Global Field Power
HF	Human Factor
HPE	Human Performance Envelope
ISA	Instant Self-Assessment
LASSO	Least Absolute Shrinkage and Selection Operator
MVAR	Multivariate Autoregressive (model)
NaN	Not a Number
OLS	Ordinary Least Squares
PID	Partial Information Decomposition
PID-LASSO	Partial Information Decomposition—Least Absolute Shrinkage and Selection Operator
PR	PageRank
rmcorr	Repeated Measures Correlation
RT	Reaction Time
SESAR	Single European Sky ATM Research
SME	Subject Matter Expert
SS	State-Space (model)
STCA	Short-Term Conflict Alert
TE	Transfer Entropy
VAR	Vector Autoregressive (model)
